# Clinical outcome and risk factors of neonatal sepsis among neonates in Felege Hiwot referral Hospital, Bahir Dar, Amhara Regional State, North West Ethiopia 2016: a retrospective chart review

**DOI:** 10.1186/s13104-017-2573-1

**Published:** 2017-07-11

**Authors:** Tilahun Tewabe, Seida Mohammed, Yibeltal Tilahun, Birhanie Melaku, Mequanint Fenta, Tsigiereda Dagnaw, Amare Belachew, Ashagre Molla, Habte Belete

**Affiliations:** College of Medicine and Health Sciences, Bahir Dar University, Bahir Dar, Ethiopia

**Keywords:** Clinical outcome, Risk factors, Neonatal sepsis, Felege Hiwot referral hospital, Bahir Dar, North west Ethiopia

## Abstract

**Background:**

Sepsis remains a major cause of morbidity and mortality among neonates. The risk factors and clinical outcomes of sepsis are poorly understood. Most cases of sepsis occurred mostly within the first week of newborns life related to perinatal risk factors. Late onset sepsis is related to hospital acquired infections which is seen after seven days of age. The purpose of this study was to assess clinical outcome and risk factors of neonatal sepsis in Felege Hiwot referral hospital Bahir Dar, North West Ethiopia.

**Results:**

Among the total 225 neonatal charts reviewed; 164 (72.9%) were age less than or equal to 7 days, and 144 (64%) were males. About 29 (12.9%) neonates were with irregular respiratory signs and 40 (17.8%) had meconium aspiration syndrome. Regarding the clinical outcome of neonatal sepsis: 189 (84%) were improved after treatment, 9 (4%) were died and 13 (5.8%) referred to other organizations for further treatment. Respiratory distress syndrome [AOR = 0.258 (0.072–0.930)] and meconium aspiration syndrome [AOR = 0.1989 (0.059–0.664)] were the determinant factors for poor outcome of neonatal sepsis.

**Conclusion:**

The clinical outcome of neonatal sepsis in Felege Hiwot referral hospital was not satisfactory. The significant risk factors for poor outcome of neonatal sepsis were respiratory distress syndrome and meconium aspiration syndrome. Recommendations to improve neonatal outcome are: performing essential newborn care for all newborns and arranging appropriate follow up until the end of neonatal period, increasing antenatal care and early detection and management of neonatal infections or problems.

## Background

Neonatal sepsis is a systemic infection occurring in infants within 28 days of life and is a major cause of morbidity and mortality in newborns [1]. According to the international pediatric consensus conference of 2001, neonatal sepsis was defined as systemic inflammatory response syndrome in the presence of or as a result of suspected or proven infection with or without accompanying bacteremia, documented by a positive blood culture in the first 28 days of life [2].

Sepsis encompasses various systemic infections of the new born such as: septicemia, meningitis, pneumonia, arthritis, osteomyelitis and urinary tract infections [3]. Neonatal sepsis is caused by both gram-positive and gram negative bacteria’s [4, 5].

Neonatal sepsis is classified into two major categories based on the time of onset: early-onset neonatal sepsis (EONS) and late onset neonatal sepsis (LONS). Early-onset neonatal sepsis appears within the first seven days of life and most cases appear within 24 h of birth. While late onset neonatal sepsis occurs after 8 days of infants life and is mostly acquired after delivery [5, 6].

Sepsis is diagnosed by: a complete white blood cell count with differential, blood culture, urine cultures, and a lumbar puncture for cell count and culture. To clear the diagnosis of early onset sepsis factors that predispose the neonate for sepsis such as maternal infection and prolonged rupture of membranes, and prematurity are also considered [1, 6].

Signs and symptoms of infection in neonates are subtle and non-specific, may present with one or more of the following: hypothermia or fever, lethargy, poor cry, refusal to suck, poor perfusion, prolonged capillary refill time, hypotonia, absent neonatal reflexes, bulging fontanel, brady/tachycardia, respiratory distress, apnea and gasping respiration, hypo/hyperglycemia, and metabolic acidosis [3, 6, 7].

Risk factors for early onset of sepsis includes premature rupture of membrane (PROM), fever, chorioamnionitis, repeated vaginal examination, meconium stained amniotic fluid, dietary intake of contaminated foods, cervical cerclage, place of birth, prematurity, low birth weight, complicated or instrument-assisted delivery, and low appearance pulse grimace activity respiration (APGAR) scores. Late onset of sepsis acquiring nosocomial infections and invasive procedures during hospital admission [1, 6, 8].

Antimicrobials used to treat sepsis are combinations and in most units are penicillin (Benzyl penicillin, Ampicillin, or Cloxacillin) together with an aminoglycoside, most commonly Gentamicin and is largely preventable by timely recognition, rational antimicrobial therapy and aggressive supportive care [3, 9].

Globally, sepsis is one of the major causes of morbidity and mortality among neonates [4], according to WHO sepsis caused approximately 12% of the 2.9 million neonatal deaths in 2012 [10]. Out these deaths 99% occur in developing countries [11].

In Africa sepsis accounts 28% neonatal deaths [12] and infectious causes accounts 68 deaths per 1000 live births [13]. In Ethiopia from prenatal mortalities sepsis covers 5% [14]. In Debrezeyt, Ethiopia the overall poor outcomes of NS were 26% including deaths [8].

Therefore the purpose of this study was to assess clinical outcomes and risk factors of neonatal sepsis in Felege Hiwot referral hospital, Bahir Dar, North West Ethiopia.

## Methods

### Study settings and period

An institution based quantitative retrospective chart review was conducted from April 30 to May 30, 2016 in Felege Hiwot referral hospital. It is located in Amhara regional state, Bahir Dar, Ethiopia. It is 565 km away from Addis Ababa. The hospital was established in April 1963 in collaboration with the Ethiopian people and the German government. The hospital has different departments that provide specialized services in outpatient, inpatient and operation theatre departments. It provides services for approximately for 130,000 populations and has more than 415 beds and gives services for the western part of Amhara region as a Referral hospital. Annually nearly 550 neonates with sepsis were admitted at Felege Hiwot referral hospital. The neonatal intensive care unit has 30 beds and there were five pediatricians and 11 nurses.

The sample size of the study was calculated using single population proportion formula by considering the following assumptions: prevalence (*P*) = 50%, confidence level (*CI*) = 95%, margin of error (*W*) = 5% and by using correction formula since the total population is below 10,000 the final calculated sample size became 225.

### Measurement

Data was collected and registered by using structured check list. The check list was prepared by reviewing different literatures done on similar topics. The check list consists of socio demographic information of mother and neonate, maternal and neonatal risk factors, and health service related factors for poor outcome of sepsis. The data were collected by four data collectors and one supervisor and finally submitted to the investigator as scheduled. Before the data collection period data collectors and supervisors were oriented and trained for a day on how to record and collect data.

### Operational definitions of the variables

- Early onset of sepsis: If sepsis is occurred from birth to 7 days of age.
- Late onset of sepsis: If sepsis is occurred between 8 and 28 days of age.
- Good outcome: If neonate is improved after completing the treatment without any complications like: seizure, meningitis, shock, deafness and blindness.
- Poor outcome: If neonate is not improved after completing the treatment, presented with complications, referred to other health institutions, died and refused against medical treatment.

## Results

### Socio demographic data

A total of 225 neonatal charts with sepsis were studied. From total 144 (64%) were males, 164 (72.9%) were age less than 7 days, 115 (51.1%) mothers were between 19 and 29 years old, and 133 (59.1%) were rural residents (Tables 1, 2).

**Table 1:** Neonatal related risk factors for sepsis in Felege-Hiwot referral hospital, North West Bahir Dar, Ethiopia, 2016

Variables	Frequency	Percent
Sex		
M	144	64
F	81	36
Age of infant		
0–7 days	164	72.9
8–28	61	27.1
Birth weight (g)		
<1500	7	3.1
<2500	71	31.6
2500–4000	143	63.6
>4000	4	1.8
Prematurity (weeks)		
<37	46	20.4
37–42	173	76.9
>42	6	2.7
Birth asphyxia		
Yes	8	3.6
No	217	96.4
Associated infection (n = 10) (4.5%)		
Meningitis	8	3.6
Hydrocephalus	2	0.9
Had resuscitation		
Yes	8	3.6
No	217	96.4
Mode of ventilation (n = 8) (3.6%)		
Ambubag	4	1.8
Suction machine	2	0.9
Ambubag and suction machine	2	0.9
APGAR score		
<3	4	1.8
4–6	69	30.7
>7	152	67.6
Birth injury		
Yes	4	1.8
No	221	98.2
BCG and polio vaccinated		
Yes	132	58.7
No	93	41.3
Immune suppressant drug		
Yes	1	0.4
No	224	99.6
Prophylaxis of HIV infection		
Yes	9	4
No	216	96
Any skin infection/umbilical stump		
Yes	6	2.7
No	219	97.3
Endotracheal intubation (n = 222)		
Yes	1	0.4
No	221	98.2
NG tube feeding (n = 218)		
Yes	54	24
No	164	72.9
IV line medication		
Gentamycin + ampicillin	203	90.2
Ceftriaxone + genta	20	8.9
Vancomycin + ceftazidim	2	0.9
Out come after admission		
Improved	189	84
Death	9	4
Referral	13	5.8
Refuse against medical treatment	14	6.2

**Table 2:** Maternal related risk factors that predisposed to neonatal sepsis during pregnancy in Felege-Hiwot referral hospital, North West Bahir Dar, Ethiopia, 2016

Variables	Frequency	Percentage
Age of the mother (years)		
<18	14	6.2
19–29	115	51.1
30–34	67	29.8
>35	29	12.9
Residence		
Rural	133	59.1
Urban	92	40.9
No. of pregnancy		
Primi gravid	97	43.1
Multi gravid	124	55.1
Grand multi Para	4	1.8
>24 h	8	3.6
ANC follow up		
Yes	214	95.1
No	11	4.9
TT vaccination		
Yes	214	95.1
No	11	4.9
UTI during pregnancy (n = 183)		
Yes	9	4
No	174	77.3
Febrile Hx of mother (n = 205)		
Yes	47	20.9
No	158	70.2
Twin pregnancy		
Yes	12	5.3
No	213	94.7
Cervical cerclage (n = 191)		
Yes	2	0.9
No	189	84
Maternal infection hx (n = 205)		
Yes	4	1.8
No	201	89.3
Place of birth		
Hospital	133	59.1
Health center	81	36
Home	11	4.9
Mode of delivery		
SVD	147	65.3
Instrumental	17	7.6
C/S	61	27.1
PROM (217)		
Yes	47	20.9
No	170	75.6
PROM > 12 h (n = 207)		
Yes	29	12.9
No	178	79.1
PROM intrapartum antibiotic (n = 203)		
Yes	43	19.1
No	160	71.1
Duration of labor (n = 174) (h)		
<8	51	22.7
8–18	69	30.7
18–24	46	20.4
Obstructed labor hx (n = 222)		
Yes	28	12.4
No	194	86.2
Chorioamnionitis hx (n = 186)		
Yes	34	15.1
No	152	67.6
Meconium hx (n = 183)		
Yes	40	17.8
No	143	63.6
Foul lochia (n = 180)		
Yes	10	4.4
No	170	75.6

### Neonatal related risk factors for sepsis

From 225 neonates 169 (75.1%) were admitted with early onset of sepsis. From total 71 (31.6%) were low birth weight, 173 (76.9%) were term (37–42 weeks), 8 (3.6%) were presented with meningitis, 8 (3.6%) had history of birth asphyxia, and 73 (32.4%) neonates were with APGAR score less than six. Most, 203 (90.2%) neonates were treated with Ampicillin and Gentamycin. About 89 (84%) were improved after completing the treatment but 9 (14%) were died (Table 1).

### Maternal related risk factor for neonatal sepsis

More than half of the mothers 124 (55.1%) were multigravida. Majority (95.1%) of mothers received ANC follow up and 9 (4%) mothers had history of urinary tract infection during their pregnancy. About 47 (20.9%) mothers were febrile, 12 (5.3%) mothers were twin delivered, 2 (0.9%) were having history of cervical cerclage and 4 (1.8%) were mothers with history medical problem during pregnancy.

One hundred and thirty-three (59.1%) mothers delivered their newborn in hospital and 61 (27.1%) mothers delivered by caesarean section. With regard to rupture of membrane, 47 (20.9%) had history of PROM and out of them 29 (12.9%) were for more than 12 h duration. Out of all mothers with PROM, antibiotic was given for 43 (19.1%) mothers. About 46 (20.4%) mothers has history of prolonged duration of labor. While 28 (12.4%), 34 (15.1%), 40 (17.8%) mothers faced obstructed labor, history of chorioamnionitis and meconium aspiration syndrome, respectively (Table 2).

### Clinical presentation of neonates with sepsis

One hundred and fifty-eight neonates (70.2%) had history of fever, and 29 (12.9%), 15 (6.7%) were history of irregular respiration and tachypnea, respectively. Majority of neonates 74 (32.9%) had poor feeding and about 43 (19.1%) had cold and clammy skin (Table 3).

**Table 3:** Clinical features of sepsis among neonates admitted in Felege-Hiwot referral hospital, North West Bahir Dar, Ethiopia, 2016

Variables	Frequency	Percentage
Have fever		
Yes	158	70.2
No	67	29.8
Respiratory features		
Tachypnea	15	6.7
Apnea	8	3.6
Hypoxia	2	0.9
Flaring or grunting	5	2.2
Irregular respiration	29	12.9
Retraction	3	1.3
No respiratory sign	143	63.6
More than one symptoms	20	8.9
Gastro intestinal features		
Poor feeding	74	32.9
Vomiting	17	7.6
Diarrhea	1	0.4
Abdominal distention	1	0.4
No symptoms	107	47.6
More than one symptom	25	11.1
Neurologic features		
Decrease activity/lethargy	10	4.4
Irritability	33	14.7
Tremors or seizure	2	0.9
No neurologic signs	180	80
Metabolic features		
Hypoglycemia	1	0.4
No metabolic sign	224	99.6
Skin color change sign		
Cold or clammy skin	43	19.1
Pallor or skin molting	5	2.2
Petechiae or purpura	3	1.3
No skin color change	174	77.3

### Diagnostic/laboratory results of neonates with sepsis

Of the total 39 samples tested for culture 39 (17.3%) were gram negative. While the CSF result showed; white blood cell (WBC) count >5 cells/µL was in 15 (6.7%) cases, 10 (4.4%) were glucose <40 mg/dL, 6 (2.7%) were protein >45 mg/dL and WBC count in CBC profile were 142 (63.1%) (Table 4).

**Table 4:** Neonatal sepsis diagnostic test results in Felege-Hiwot referral hospital, North West Bahir Dar, Ethiopia, 2016

Variables	Frequency	Percent
Culture and gram stain result		
Gram negative	39	17.3
Not done	186	82.7
Appearance of CSF (20) (8.9%)		
Clear	10	4.4
Cloudy	6	2.7
Bloody	4	1.8
Lumbar puncture result about WBC (20) (8.9%)		
0–5 cells/µL	5	2.2
>5 cells/µL	15	6.7
Glucose (20) (8.9%)		
<40 mg/dL	10	4.4
>40 mg/dL	10	4.4
Protein (20) (8.9%)		
<45 mg/dL	6	2.7
>45 mg/dL	14	6.2
Gram stain (20) (8.9%)		
Gram negative	20	8.9
WBC result in CBC profile		
<4 billion cells/L	5	2.2
5–10.5 billion cells/L	54	24
>10.5 billion cells/L	142	63.1
No CBC profile	24	10.7
X-ray result		
Normal finding	5	2.2
No X-ray	220	97.8

### Factors associated with clinical outcome of neonatal sepsis

First variables were tested by using bivariate analysis. Variables which were associated (*p* < 0.05) in the bivariate analysis were tested in the in the final multivariate analysis to see their significant association with poor outcome of neonatal sepsis. The independent predictor of poor outcome of neonatal sepsis were; respiratory distress syndrome and history of meconium aspiration syndrome.

Respiratory distress syndrome was significantly associated with poor outcome of neonatal sepsis. Those neonates with respiratory distress syndrome were 74.2% more likely to develop poor outcome (AOR 0.258: 0.072, 0.930) than neonates without respiratory distress syndrome.

Meconium aspiration syndrome was significantly associated with poor outcome of sepsis. Neonates with meconium aspiration syndrome were 80.2% more likely to develop poor neonatal outcome (AOR 0.198: 0.059, 0.664) than neonates without history of meconium aspiration syndrome (Table 5).

**Table 5:** Factors associated with clinical outcome of neonatal sepsis in Felege-Hiwot referral hospital, North West Bahir Dar, Ethiopia, 2016

Variables	Clinical outcomes		COR	AOR	*P* value
	Good	Poor			
Birth weight (g)					
<2500	60 (76.9%)	18 (23.1%)	0.465 (0.226–0.957)		
>2500	129 (87.8%)	18 (12.2%)	1		
Asphyxia					
Yes	3 (37.5%)	5 (62.5%)	1 (0.023–0.440)		
Respiratory distress					
No	186 (85.7%)	31 (14.3%)	1		
Yes	129 (90.2%)	14 (9.8%)	0.296 (0.142–0.618)	*0.258 (0.072–0.930)*	*0.038*
No	60 (73.2%)	22 (26.8%)	1		
Skin color					
Good	153 (87.9%)	21 (12.1%)	0.329 (0.155–0.701)		
Poor	36 (70.6%)	15 (29.4%)	1		
APGAR score					
<6	61 (83.6%)	12 (16.4%)	0.953 (0.447–2.032)		
>7	128 (84.2%)	24 (15.8%)	1		
Onset of illness					
Early	147 (87%)	22 (13%)	2.227 (1.049–4.728)		
Late	42 (75%)	14 (25%)	1		
Iv line medications					
Gentamycin + ampicillin	175 (86.2%)	28 (13.8%)	3.571 (1.373–9.289)		
Ceftriaxone + Gentamycin	14 (63.6%)	8 (36.4%)	1		
Place of birth					
Health institution	182 (85%)	32 (15%)	0.308 (0.085–1.112)		
Home	7 (63.6%)	4 (36.4%)	1		
Maternal fever					
Yes	45 (95.7%)	2 (4.3%)	4.846 (1.110–21.163)		
No	130 (82.3%)	28 (17.7%)	1		
NG tube feeding					
Yes	53 (98.1%)	1 (1.9%)	13.862 (1.850–103.87)		
No	130 (79.3%)	34 (20.7%)	1		
Meconium aspiration					
Yes	27 (67.5%)	13 (32.5%)	0.299 (0.131–0.683)	*0.198 (0.059–0.664)*	*0.009*
No	125 (87.4%)	18 (12.6%)	1		

Italic value indicates p value less than <.05

## Discussion

Neonatal sepsis is a systemic infection occurring in infants at less than 28 days of life and is an important cause of morbidity and mortality of newborns [1]. It encompasses various systemic infections of the new born such as septicemia, meningitis, pneumonia, arthritis, osteomyelitis and urinary tract infections [3].

Risk factors for early onset of sepsis includes: premature rupture of membrane, fever, chorioamnionitis, repeated vaginal examination, meconium stained amniotic fluid, dietary intake of contaminated foods, cervical cerclage, place of birth, prematurity, low birth weight, complicated or instrument assisted delivery, and low appearance pulse grimace activity respiration (APGAR) scores. Late onset of sepsis acquiring nosocomial infections and invasive procedures during hospital admission [1, 6, 8].

In this study 84% of neonates had good outcome after treatment. This result comparable with a study done in Debrezeyt, Ethiopia favorable outcome of neonatal sepsis were 74% [8] and in Jimma neonatal death due to infections was 34.3% [15], in other studies done in Ethiopia mortalities due to sepsis accounts 5% a hospital based data [14], in Uganda death rates associated with sepsis was 18.1% [16], in Sudan neonatal mortality due to sepsis was found to be 14.5% [13], Egypt mortality rate of neonatal sepsis were 51% for early onset sepsis and 42.9% for late onset sepsis [4], Iran neonatal sepsis was estimated at 27.4% [17], and Latin America: Brazil, Colombia and Mexico mortality rate of neonatal sepsis were 56, 36 and 28% respectively [18].

In this study respiratory distress syndrome was identified as the determinant factor for poor clinical outcome neonatal sepsis. Neonates with history of respiratory distress syndrome were 74.2% more likely to develop poor neonatal outcome. This result comparable with studies done Uganda [16] in which tachypnea (AOR 1.07: 0.65, 1.77) was the determinant factor for poor outcome of sepsis, and in Sudan [19] where tachypnea results 69.4% for poor outcome of sepsis. This was due to health workers ignorance the syndromes, poor early detection of signs and due to the mothers delay to come in health institution.

Meconium aspiration syndrome history was significantly associated with clinical outcome of sepsis. Neonates with meconium aspiration syndrome history were 80.2% more likely to develop poor outcome. Which is similar with a study in Uganda [16] where neonates with meconium aspiration syndrome were 2.5 times more likely to develop poor outcome than neonates without history of meconium aspiration. This is showed that after meconium aspiration strict follow up is needed. This may be due to health workers poor neonatal performance skill and ignorance of meconium aspiration signs.

## Conclusion

In this study the favorable outcomes of neonatal sepsis was 189 (84%). The determinant factors for poor outcome of neonatal sepsis were respiratory distress syndromes and meconium aspiration syndrome. Recommendations to improve neonatal outcome are: performing essential newborn care for all newborns and arranging appropriate follow up until the end of neonatal period and early detection and management of neonatal infections or problems.

## Abbreviations

ANC: ante natal care
APGAR: appearance pulse grimace activity respiration
AOR: adjust odd ratio
CI: confidence interval
CS: cesarean section
EONS: early onset neonatal sepsis
FHRH: Felege Hiwot referral hospital
LBW: low birth weight
LONS: late onset neonatal sepsis
NGO: non governmental organization
PROM: premature rupture of membrane
SPSS: statistical Package for social science
WHO: World Health Organization
